# Supplementary material for: Single methyl groups can act as toggle switches to specify transmembrane Protein-protein interactions
Source: eLife. 2017 Sep 4;6:e27701. doi: 10.7554/eLife.27701 (PMC5597333; doi:10.7554/eLife.27701)
Supplement: Transparent reporting form [file elife-27701-transrepform.pdf]

eLIFE
1st Floor
24 Hills Road
Cambridge CB2 1JP, UK
P 01223 855340
W elifesciences.org
T @elife

eLife's transparent reporting form

We encourage authors to provide detailed information *within their submission* to facilitate the interpretation and replication of experiments. If you have any questions, please contact us: editorial@elifesciences.org.

Sample-size estimation

- You should state whether an appropriate sample size was computed when the study was being designed
- You should state the statistical method of sample size computation and any required assumptions
- If no explicit power analysis was used, you should describe how you decided what sample (replicate) size (number) to use

Please outline where this information can be found within the submission (e.g., page numbers or figure legends), or explain why this information doesn't apply to your submission:

Not applicable.

Replicates

- You should report how often each experiment was performed
- You should include a definition of biological versus technical replication
- The data obtained should be provided and sufficient information should be provided to indicate the number of independent biological and/or technical replicates
- If you encountered any outliers, you should describe how these were handled
- Criteria for exclusion/inclusion of data should be clearly stated
- High-throughput sequence data should be uploaded before submission, with a private link for reviewers provided (these are available from both GEO and ArrayExpress)

Please outline where this information can be found within the submission (e.g., page numbers or figure legends), or explain why this information doesn't apply to your submission:

All IL-3 tests were performed in three independent biological replicates (i.e. independent infections to express traptamers followed by cell counts after IL-3 removal). All reported experiments included positive and negative controls that performed as expected, and no outliers in these experiments were excluded. This is described in the Methods (Cells, retrovirus infection, and activity testing section) and in the legends to the following figures:
Figure 2A, 2B, 2C, 2D
Figure 5
Figure 6B, 6C (left panel), 6D, 6E (left panel)

Figures showing biochemical experiments are representative of independent experiments conducted at least three times
Figure 3A, 3B, 3C, 3D
Figure 6C (right panel) and 6E (right panel)

In all replicate NMR experiments on different batches of peptides, the chemical shift differences induced by the traptamers did not vary more than a maximum of 0.04 ppm between data sets. This is stated in the Methods (NMR spectroscopy and titrations section).

Statistical reporting

- Statistical analysis methods should be described and justified
- Raw data should be presented in figures whenever informative to do so (typically when N per group is less than 10)
- For each experiment, you should identify the statistical tests used, exact values of N, definitions of center, methods of multiple test correction, and dispersion and precision measures (e.g., mean, median, SD, SEM, confidence intervals; and, for the major substantive results, a measure of effect size (e.g., Pearson's r, Cohen's d)
- Report exact p-values wherever possible alongside the summary statistics and 95% confidence intervals. These should be reported for all key questions and not only when the p-value is less than 0.05.

Please outline where this information can be found within the submission (e.g., page numbers or figure legends), or explain why this information doesn't apply to your submission:

All IL-3 tests were performed in three independent biological replicates (i.e. independent infections to express traptamers followed by cell counts after IL-3 removal). All reported experiments included positive and negative controls that performed as expected, and no outliers in these experiments were excluded. All graphs show average values for IL-3 tests +/- SEM. Statistical significance of difference between control and experimental samples were evaluated by two-tailed Student's t-tests with unequal variance, performed using T.TEST function in Microsoft Excel (2013). Significance cut-off for differences in cell numbers was established by p value <0.05. Source data are reported in Source files for Figures 2, 5, 6.

eLife Sciences Publications, Ltd is a limited liability non-profit non-stock corporation incorporated in the State of Delaware, USA, with company number 5030732, and is registered in the UK with company number FC030576 and branch number BR015634 at the address 1st Floor, 24 Hills Road, Cambridge CB2 1JP | August 2014

(For large datasets, or papers with a very large number of statistical tests, you may upload a single table file with tests, Ns, etc., with reference to page numbers in the manuscript.)

Additional data files ("source data")

- We encourage you to upload relevant additional data files, such as numerical data that are represented as a graph in a figure, or as a summary table
- Where provided, these should be in the most useful format, and they can be uploaded as "Source data" files linked to a main figure or table
- Include model definition files including the full list of parameters used
- Include code used for data analysis (e.g., R, MatLab)
- Avoid stating that data files are "available upon request"

Please indicate the figures or tables for which source data files have been provided:

Additional data files are uploaded as source data for Figures 2, 5, and 6.
